# Relationship of handgrip strength, asymmetries, and calf circumference with functional capacity in individuals with intellectual disabilities: an age group analysis

**DOI:** 10.1186/s13102-025-01181-8

**Published:** 2025-07-02

**Authors:** Justine Mendoza-Puelma, Julio B. Melo, Gerson Ferrari, Paloma Ferrero-Hernández, Alexis Espinoza-Salinas, Pedro Valdivia-Moral, Antonio Castillo-Paredes, Emilio Jofré-Saldía, Claudio Farías-Valenzuela

**Affiliations:** 1 Escuela de Ciencias de la Actividad Física, el Deporte y la Salud, Universidad de Santiago de Chile (USACH), Santiago, Chile; 2 Escuela de Educación Física, Pontificia Universidad Católica de Valparaíso, Valparaíso, Chile; 3 Facultad de Ciencias de la Salud, Universidad Autónoma de Chile, Providencia, Santiago, Chile; 4 Vicerrectoría de Investigación e Innovación, Universidad Arturo Prat, Iquique, Chile; 5 Facultad de Salud, Escuela de Kinesiología, Universidad Santo Tomás, Santiago, Chile; 6 Department of Didactics of Musical, Plastic and Body Expression, University of Granada, Granada, 18011 Spain; 7 Facultad de Ciencias de la Rehabilitación y Calidad de Vida, Universidad San Sebastián, Santiago, Chile

**Keywords:** Handgrip strength, Calf circumference, Muscle asymmetries, Functional capacity, Intellectual disabilities

## Abstract

**Background:**

Handgrip strength (HGS) and calf circumference (CC) are key health markers associated with dynapenia and autonomy in the general population. However, their association with functional capacity in individuals with intellectual disabilities (ID) remains unclear. This study aimed to determine the relationship between absolute and relative HGS, upper-limb strength asymmetries, and CC with functional capacity in individuals with ID.

**Methods:**

A total of 102 individuals ([31 children: mean age = 9.58 years, SD:1.82]; [30 adolescents: mean age = 14.67 years, SD: 1.34]; [41 adults: mean age = 23.56 years, SD: 5.59]) with mild to moderate ID, from four special education schools in Santiago, Chile, were assessed. HGS and asymmetries were evaluated using dynamometry, while CC was measured anthropometrically. Functional capacity was assessed using the timed up and go (TUG), 5-repetition sit-to-stand (5R-STS), 4 × 10 m agility, and countermovement jump (CMJ) tests. Pearson, Spearman, and linear regression analyses, were applied to examine the relationships.

**Results:**

The mean values for absolute HGS, relative HGS, absolute asymmetry, percentage asymmetry, and CC were 20.71 kg, 0.35, 1.39 kg, 13.61%, and 33.89 cm, respectively. Functional test averages were 6.51 s (TUG), 10.46 s (5R-STS), 19.43 s (4 × 10 m agility), and 12.47 cm (CMJ). Significant correlations were found between absolute and relative HGS with all functional tests across age groups. Absolute HGS asymmetries correlated with some functional tests in children and adolescents, while percentage asymmetry and CC showed no associations. The very high age-group-specific correlations were TUG (*r* = -0.73; β = -0.34; R^2^ = 0.66) in children, agility 4 × 10 m test (*r* = -0.73; β = -0.26; R^2^ = 0.66) in adolescents, and CMJ (*r* = 0.71; β = 27.30; R^2^ = 0.71) in adults.

**Conclusions:**

Absolute and relative HGS, as well as absolute asymmetries, are key predictors of functional capacity in individuals with ID. Implementing strength training interventions from an early school age is critical to preserving functional capacity in this population.

**Clinical trial:**

Not applicable.

## Background

Intellectual disability (ID) is a lifelong neurodevelopmental disorder characterized by cognitive impairment, leading to difficulties in adapting to and interacting with the environment and peers [1]. ID affects an estimated 1–3% of the population, with approximately 85% of cases classified as mild ID. Additionally, the prevalence of ID is about 1.5 times higher in men than in women [2]. The causes of ID are linked to hereditary factors, nutritional deficiencies, and chromosomal mutations [3, 4], which often result in premature functional decline [5]. Individuals with ID are at an increased risk of overweight and obesity. These conditions, combined with predominantly sedentary behaviors [5, 6] contribute to reduced muscular and cardiorespiratory fitness, thereby elevating the risk of cardiometabolic diseases [6].

High muscle strength is linked to a reduced risk of cardiometabolic diseases and mortality [7], with handgrip strength (HGS) - both absolute (HGS-A) and relative (HGS-R)– widely recognized as predictors of overall health [8, 9]. Notably, HGS-R has shown a stronger inverse correlation with adiposity indicators compared to HGS-A in a sample of schoolchildren. A study by Farías-Valenzuela et al. [10] found that higher levels of HGS-A and trunk extensor strength were associated with improved functional capacity in field tests among adolescents with ID. However, there is no clarity on how HGS-A and HGS-R could be related to the functional capacity of individuals with ID from other age groups.

McGrath et al. [11] reported that functional impairments in daily living activities are associated with percentage differences in HGS between upper limbs, defining HGS asymmetry as a percentage difference greater than 10%. Similarly, greater asymmetries in lower-limb strength, measured through knee extension, were linked to slower gait speed. Furthermore, HGS asymmetries, combined with lower HGS, lower levels of voluntary muscle activation [12] were associated with functional impairments, with this combination significantly increasing the likelihood of disability in daily living activities [13, 14] and multimorbidity’s [15]. However, scientific evidence on the study of asymmetries in individuals with ID and their impact on functionality is scarce.

For its part calf circumference (CC) is an anthropometric measure closely associated with higher physical performance [16, 17]. It has been widely used as a marker for detecting sarcopenia, with reductions in CC positively linked to cognitive decline [18]. According to Wang et al. [16] CC can serve as a predictor of muscle strength and physical performance, showing a positive correlation with gait speed. Additionally, CC has been identified as an independent risk factor related to physical performance, functionality, and frailty in a sample of older adults [17].

Functionality is intrinsically linked to autonomy, independence, and quality of life [19]. Individuals with ID encounter numerous factors that negatively impact their functionality, including significantly lower physical fitness levels compared to those with typical development [20]. Enhancing functional capacity can promote greater independence and autonomy in daily activities, reduce the risk of falls, and alleviate challenges associated with performing vocational and social tasks [21].

Despite prior research, evidence on functional capacity levels and their relationship with muscle strength in individuals with ID remains limited. Specifically, the associations between HGS, asymmetries, and CC as predictors of functionality in Chilean children, adolescents, and adults with ID are not well understood. We hypothesized that higher absolute and relative HGS and better CC will be positively associated with functional capacity, while greater upper-limb strength asymmetries will be negatively associated with functional capacity in individuals with ID across age groups. Therefore, this study aimed to examine the associations between absolute and relative HGS, upper-limb strength asymmetries, and CC with the functional capacity of individuals with ID across different age groups.

## Methods

### Study design and sample

This correlational cross-sectional study involved 102 individuals with ID (54.9% males) with a mean age of 16.7 years (SD = 7.05). Participants were divided into three groups: (i) children (6–12 years), (ii) adolescents (13–17 years), and (iii) adults (18–26 years). They were recruited from four special education institutions in Santiago, Chile. A convenience sampling method was employed due to the research team’s accessibility to this specific group of students, facilitated by a prior agreement between the university and the schools for practical activities and projects [22].

To determine the statistical power based on the sample size of 102 participants, a post-hoc power analysis was conducted using G-Power version 3.1. The analysis was based on an association test within the F family. An alpha level of 0.05, a moderate effect size (f² = 0.15), and 10 predictor variables were used. The results indicated that, given the sample size, the study achieved a statistical power (1-β) of 0.94.

Inclusion criteria included: a diagnosis of mild to moderate ID (IQ ≤ 69 − 50 and ≤ 49 − 35, respectively) based on the Wechsler Intelligence Scale for Children < 16 years (WISC-III) [23], or the Wechsler Adult Intelligence Scale (WAIS-IV) [24] for those aged ≥ 16 years, with and without syndromes associated with ID, as assessed by a psychologist at each educational institution; independent mobility; regular participation in physical education classes at the institution (sessions lasting between 90 and 120 min, with a frequency of once or twice per week, performed on alternate days); and a valid medical certificate confirming eligibility. Exclusion criteria were: a diagnosis of severe-profound ID (IQ < 35); dependence on motor tasks; incomplete muscle strength or functional tests; or reliance on a wheelchair.

This study adhered to the principles of the Declaration of Helsinki and was approved by the ethics committee of the University of Granada (approval code: 2052/CEIH/2021). During this meeting, parents and tutors reviewed and signed the informed consent form, ensuring students’ voluntary and anonymous participation, with the option to withdraw at any time. Written informed consent obtained from all those ≥ 16 years of age and also from legal guardians/parents of participants < 16 years of age, before commencement of the study.

### Procedures

Data collection was carried out between August and September 2021, and the data belong to the LUDOINCLUSIÓN ^®^ project. Initial communication with the directors of four special education centers occurred between June and July. A meeting was held with tutors to explain the intervention, including its objectives and evaluation protocols. During this meeting, parents and tutors reviewed and signed the informed consent form, ensuring students’ voluntary and anonymous participation, with the option to withdraw at any time. Evaluations were scheduled for both morning and afternoon sessions and were organized into three stations, each staffed by a minimum of two and a maximum of four evaluators. Students were accompanied by a tutor, who provided support as needed. Detailed procedures have been previously published [5].

At the first station, anthropometric measurements were collected, including body weight, height, waist circumference, calf circumference (CC), body mass index (BMI), and waist-to-height ratio (WHtR). The second station assessed muscle strength using handgrip strength tests (HGS-A and HGS-R). Finally, at the third station, functional capacity was assessed using the timed up and go (TUG) test, the five repetition sit-to-stand test (5R-STS), the 4 × 10 m agility test, and the countermovement jump (CMJ). Data were categorized by participants’ age group.

### Handgrip strength and asymmetries

HGS was measured in kilograms using a hydraulic dynamometer (Baseline^®^ model LiTE^®^, Fabrication Enterprises, Inc., New York, NY, USA) in accordance with the guidelines of the American College of Sports Medicine (ACSM) [25]. Participants received verbal instructions and visual examples of the test, which required exerting maximal force. Three alternate attempts were made for each hand [26], with the first attempt being familiarization. The average of the second and third attempts was considered the final measure of HGS-A. A 1-minute rest period was given between attempts.

Once HGS-A was obtained, relative handgrip strength (HGS-R) was calculated using the formula: (HGS-A (kg) / body weight (kg)) [27]. Based on the average HGS-A for each hand, absolute handgrip asymmetry was calculated using the formula: proposed by Bishop et al. [28]: (Stronger hand - weaker hand / stronger hand) x 100.

### Anthropometry and calf circumference

Body weight and height were measured using a digital scale with an integrated stadiometer (SECA Mod 769^®^, Hamburg, Germany). Calf and waist circumference were assessed using a non-elastic metal tape measure (CESCORF^®^, Porto Alegre, Rio Grande do Sul, Brazil), calibrated in centimeters with millimeter precision. BMI and WHtR were subsequently calculated.

CC was measured at the point of greatest circumference of the right calf, in a plane perpendicular to its longitudinal axis, with the participant standing upright both feet on the ground, following the method described by Tejero-González et al. [29].

### Functional capacity

#### Timed up and go test

This test required marking a distance of 3 m, with a chair positioned at one end and an obstacle at the other. Participants were given verbal and visual instruction to stand up from the chair, walk as quickly as possible to the obstacle, circle around it, and return to sit in the chair. The time taken for this task was recorded in seconds.

Before performing the test, each participant sat with their feet on the ground and their back against the chair, as described by Martín et al. [30]. Three attempts were performed, with the first serving as familiarization. The average of the two subsequent attempts was recorded as the final result.

### Five times sit to stand test

This test involved a 43.cm highchair, where the participants performed five consecutive sit-to-stand cycles as quickly as possible. The test began with the participant seated, arms crossed over their chest, and feet flat on the floor. Three attempts were performed, the first was for familiarization, and the best time between the second and third attempts was recorded in seconds [31].

### Agility test (4 × 10 m)

This test required marking a 10-meter distance, where the participants were required to run back and forth four times as quickly as possible. An evaluator was positioned at each end to record the time in seconds and provide encouragement to ensure participants completed the test. The first attempt was for familiarization, while the second was considered the final measurement, recorded in seconds. This protocol was adapted from the Alpha Fitness Test battery, and has been validated for individuals with ID [29].

### Countermovement jump

Jump height was measured using a contact platform (Chronojump-Boscosystem^®^, Barcelona, Spain). Participants stood with hands on their hips, performed a knee flexion-extension, and jumped to reach the greatest height possible. A familiarization test was performed before the official evaluation. Each participant executed three jumps, the first one was for familiarization, while the average of the second and third attempts was recorded as the final result [32].

### Statistical analysis

The normality of the sample was assessed using the Kolmogorov-Smirnov test, while the Shapiro-Wilk test was applied to evaluate normality within each age group. Descriptive statistics were presented as mean and standard deviation. A one-way ANOVA and Kruskal Wallis test were used to compare differences in the analyzed variables among children, adolescents, and adults. To examine correlations between HGS, HGS asymmetries, calf circumference, and functional capacity tests across age groups, Pearson and Spearman coefficients were used. Correlation values (r) were interpreted as follows: trivial (< 0.09), small (0.10–0.29), moderate (0.30–0.49), high (0.50– 0.69), very high (0.70–0.89) and almost perfect (> 0.90) [33].

Additionally, a univariate linear regression model beta was employed to predict a/the relationship between functional tests and strength indicators. Prediction values are presented as: β-value (β), confidence interval (CI 95%) and coefficient of determination (R^2^). All statistical analyses were performed using SPSS version 26.0 (SPSS Inc., IBM Corp., Armonk, New York, NY, USA). A significance level of 5% was set for all analyses.

## Results

The total sample included 102 individuals: 31 children (31.4%), 30 adolescents (29.4%), and 41 adults (40.2%). Significant differences (*p* < 0.05) were observed in all anthropometric measurements among different age groups of individuals with ID (Table 1).

**Table 1:** Anthropometric characteristics of individuals with intellectual disabilities, divided by age group

Variables		Total (*n* = 102)	Children (*n* = 31)	Adolescents (*n* = 30)	Adults (*n* = 41)	*p*-value
**Age (Years)**	Mean (SD)	16.70 (7.05)	9.58 (1.82)	14.67 (1.34)	23.56 (5.59)	< 0.001^a*^
	Median (IQR)	15.00 (11.00–21.00)	10.00 (8.00–11.00)	15.00 (13.00–16.00)	22.00 (19.50–26.50)	
**Weight (Kg)**	Mean (SD)	61.25 (22.19)	43.93 (17.07)	67.20 (21.12)	69.98 (19.08)	< 0.001^b*^
	Median (IQR)	60.00 (44.00–76.00)	40.00 (29.00–58.00)	67.50 (48.50-79.25)	68.00 (54.00-81.50)	
**Height (m)**	Mean (SD)	1.54 (0.11)	1.41 (0.12)	1.64 (0.11)	1.58 (0.11)	< 0.001^b*^
	Median (IQR)	1.54 (1.44–1.67)	1.41 (1.34–1.49)	1.67 (1.55–1.72)	1.59 (1.51–1.66)	
**BMI (Kg/m2)**	Mean (SD)	24.94 (8.05)	20.72 (6.60)	24.75 (6.82)	28.26 (8.50)	< 0.001^b*^
	Median (IQR)	24.33 (19.16–28.80)	20.64 (16.09–26.25)	23.94 (19.91–28.12)	27.37 (22.47–32.67)	
**WC (cm)**	Mean (SD)	80.48 (16.96)	71.45 (13.95)	79.69 (16.97)	87.88 (15.87)	< 0.001^b*^
	Median (IQR)	80.35 (68.00-90.42)	69.50 (58.50–80.20)	80.55 (67.40-89.35)	86.00 (77.65–94.55)	
**WHtR**	Mean (SD)	0.52 (0.11)	0.48 (0.12)	0.48 (0.10)	0.55 (0.11)	< 0.001^a*^
	Median (IQR)	0.51 (0.44–0.56)	0.50 (0.42–0.56)	0.48 (0.42–0.53)	0.55 ( 0.49–0.60)	
**CC (cm)**	Mean (SD)	33.89 (5.11)	30.95 (4.91)	34.81 (5.14)	35.44 (4.35)	< 0.001^b*^
	Median (IQR)	34.55 (29.55–37.50)	29.50 (26.50–36.00)	35.60 (29.92–38.25)	34.90 (31.90-39.65)	

The data are presented as mean and standard deviation; median and interquartile range (p25-p75). BMI: Body Mass Index; WC: Waist Circumference; WHtR: Waist-to-Height Ratio; CC: Calf Circumference. *: Significance level *p* < 0.05, ^a^: Kruskal Wallis; ^b^:One-way ANOVA

Regarding muscle strength, the HGS-A were:10.68, 29.41, and 21.93 kg; HGS-R: 0.26, 0.46, and 0.33; while HGS-A asymmetry values were 1.21, 1.89, and 1.12 kg for the groups of children, adolescents, and adults, respectively.

For functional capacity, the average values were as follow: TUG: 6.84, 5.66, and 6.88 s; 5R-STS: 11.47, 8.66, and 11.00 s; 4 × 10 m agility: 21.68, 16.79, and 19.66 s; and CMJ: 10.74, 16.24, and 11.03 cm across the groups of children, adolescents, and adults, respectively. Significant differences (*p* < 0.05) were observed in HGS-A, and HGS-R, with higher values recorded in the adolescent group. However, no significant differences (*p* > 0.05) were found in HGS-A asymmetry between groups.

In terms of functional capacity, significant differences (*p* < 0.05) were noted in the 5R-STS test, the 4 × 10 m agility test, and the CMJ, with adolescents demonstrating better performance compared to the children and adult groups. No significant differences (*p* > 0.05) were found between groups for the TUG test (Table 2).

**Table 2:** Muscle strength and functional capacity characteristics of individuals with intellectual disabilities, divided by age group

Variables		Total (*n* = 102)	Children (*n* = 31)	Adolescents (*n* = 30)	Adults (*n* = 41)	*p*-value
**Muscle Strength**						
**R-HGS-A (kg)**	**Mean (SD)**	21.10 (12.31)	11.22 (6.40)	29.97 (11.94)	22.09 (10.65)	< 0.001^a^*
	**Median (IQR)**	19.25 (10.87–31.50)	10.50 (6.50–16.00)	32.00 (20.37–39.25)	21.00 (12.75–31.25)	
**L-HGS-A (kg)**	**Mean (SD)**	20.52 (12.03)	10.85 (6.02)	28.85 (10.97)	21.75 (11.19)	< 0.001^a^*
	**Median (IQR)**	18.50 (11.37–28.37)	11.50 (7.00-14.50)	30.00 (21.37–34.62)	20.50 (12.00–28.00)	
**HGS-A**	**Mean (SD)**	20.70 (12.08)	10.68 (5.76)	29.41 (11.17)	21.93 (10.82)	< 0.001^a^*
	**Median (IQR)**	18.37 (11.00-30.12)	10.75 (6.75–14.75)	30.75 (21.93–36.81)	21.25 (12.25–29.75)	
**HGS-R**	**Mean (SD)**	0.34 (0.18)	0.25 (0.13)	0.46 (0.20)	0.32 (0.16)	< 0.001^b^*
	**Median (IQR)**	0.31 (0.21–0.46)	0.25 (0.14–0.34)	0.44 (0.30–0.59)	0.31 (0.18–0.42)	
**Asymmetry HGS-A (Kg)**	**Mean (SD)**	1.39 (1.63)	1.27 (2.06)	1.89 (1.79)	1.12 (0.98)	0.13^a^
	**Median (IQR)**	1.00 (0.50-2.00)	0.50 (0.25–1.75)	1.79 (0.50.2.62)	1.00 (0.50–1.62)	
**Asymmetry HGS-A (%)**	**Mean (SD)**	13.60 (15.49)	16.65 (17.62)	12.89 (15.79)	11.82 (13.49)	0.41^a^
	**Median (IQR)**	9.60 (4.12–18.18)	10.71 (4.00-26.31)	8.34 (3.83–16.48)	8.98 (4.25–16.17)	
**Functional Capacity**						
**TUG (s)**	**Mean (SD)**	6.50 (2.56)	6.84 (2.99)	5.66 (1.97)	6.88 (2.52)	0.09^a^
	**Median (IQR)**	8.98 (7.58–11.75)	5.42 (4.60–8.96)	4.93 (4.38–7.02)	6.33 (5.43–8.27)	
**5R-STS (s)**	**Mean (SD)**	10.45 (4.57)	11.47 (6.13)	8.66 (2.27)	11.00 (4.18)	0.03^a^*
	**Median (IQR)**	8.98 (7.58–11.75)	8.88 (7.48–13.55)	8.41 (6.96–10.78)	9.74 (8.16–12.67)	
**Agility 4 × 10 m (s)**	**Mean (SD)**	19.42 (6.45)	21.68 (6.44)	16.79 (4.33)	19.66 (7.16)	0.01^a^*
	**Median (IQR)**	17.92 (14.78–21.37)	20.23 (16.66–25.69)	15.89 (13.78–18.01)	18.00 (14.59–22.25)	
**CMJ (cm)**	**Mean (SD)**	12.47 (6.78)	10.74 (5.99)	16.24 (7.22)	11.03 (6.02)	< 0.001^a^*
	**Median (IQR)**	11.42 (7.62–17.59)	10.57 (5.71–14.38)	14.25 (10.73–22.35)	9.74 (8.16–12.67)	

The data are presented as mean and standard deviation; median and interquartile range (IQR: p25-p75). R-HGS-A: Absolute Right Handgrip Strength; L-HGS-A: Absolute Left Handgrip Strength; HGS-A: Absolute Handgrip Strength; HGS-R: Relative Handgrip Strength; TUG: Timed Up and Go; 5R-STS: Five-Repetition Sit-to-Stand Test; CMJ: Countermovement Jump. *:Significance level *p* < 0.05, ^a^: Kruskal Wallis; ^b^:One-way ANOVA

HGS-A and HGS-R were significantly correlated (*p* < 0.05) with functional tests such as TUG, 5R-STS, 4 × 10 m agility, and CMJ across the groups of children, adolescents, and adults. Very high correlations were observed between HGS-A and the TUG test (*r* = -0.73) in children, the 4 × 10 m agility test (*r* = -0.73) in adolescents, and the CMJ (*r* = 0.70) in adults. Meanwhile, HGS-R demonstrated high correlations with the 4 × 10 m agility test (*r* = -0.65) and the 5R-STS test (*r* = -0.60) in children and adolescents. In adults, a very high correlation was noted between HGS-R and the CMJ (*r* = 0.71).

HGS-A showed moderate correlations with the TUG test (*r* = -0.36) in children, high correlations with the 4 × 10 m agility test (*r* = -0.50) in adolescents, and moderate correlations with the CMJ (*r* = 0.37) in adults. Percentage asymmetry of HGS-A displayed only moderate correlations (*r* = 0.32) in the adult group. Finally, CC was not correlated (*p* > 0.05) with any functional test in any of the age-defined groups. Additional correlations between HGS, HGS-A asymmetries, and CC with functional capacity are presented in Table 3.

**Table 3:** Correlation between hand grip strength, hand grip strength asymmetries, and calf circumference with the functional capacity of schoolchildren with intellectual disabilities according to age group

Functional capacity	HGS-A		HGS-*R*		Asymmetry		Asymmetry		CC	
	(kg)				HGS-A		HGS-A		(cm)	
					(Kg)		(%)			
	*r*	*p*-value	*r*	*p*-value	*r*	*p*-value	*r*	*p*-value	*r*	*p*-value
**Total (** ***n*** ** = 102)**										
**TUG (s)**	-0.46^b^	< 0.001*	-0.45^b^	< 0.001*	-0.25^b^	0.01*	-0.10^b^	0.29	-0.22^b^	0.02*
**5R-STS (s)**	-0.43^b^	< 0.001*	-0.49^b^	< 0.001*	-0.14^b^	0.14	0.03^b^	0.74	-0.13^b^	0.19
**Agility 4 × 10 m (s)**	-0.65^b^	< 0.001*	-0.55^b^	< 0.001*	-0.34^b^	< 0.001*	-0.05^b^	0.56	-0.15^b^	0.11
**CMJ (cm)**	0.61^b^	< 0.001*	0.65^b^	< 0.001*	0.34^b^	< 0.001*	0.04^b^	0.65	-0.08^b^	0.39
**Children (** ***n*** ** = 31)**										
**TUG (s)**	-0.73^b^	< 0.001*	-0.53^b^	< 0.001*	-0.38^b^	0.03*	-0.23^b^	0.21	-0.26^b^	0.15
**5R-STS (s)**	-0.38^b^	0.03*	-0.45^b^	0.01*	-0.10^b^	0.57	-0.06^b^	0.74	-0.08^b^	0.64
**Agility 4 × 10 m (s)**	-0.66^b^	< 0.001*	-0.65^b^	< 0.001*	-0.21^b^	0.25	-0.04^b^	0.80	0.11^b^	0.52
**CMJ (cm)**	0.64^b^	< 0.001*	0.62^b^	< 0.001*	0.29^b^	0.11	0.13^b^	0.45	-0.21^a^	0.25
**Adolescents (** ***n*** ** = 30)**										
**TUG (s)**	-0.64^b^	< 0.001*	-0.47^b^	< 0.001*	-0.22^b^	0.22	-0.02^b^	0.88	-0.16^b^	0.37
**5R-STS (s)**	-0.56^a^	< 0.001*	-0.60^a^	< 0.001*	-0.35^b^	0.05	-0.16^b^	0.39	-0.06^b^	0.74
**Agility 4 × 10 m (s)**	-0.73^b^	< 0.001*	-0.43^b^	0.01*	-0.50^b^	< 0.001*	-0.29^b^	0.11	-0.34^b^	0.05
**CMJ (cm)**	0.59^a^	< 0.001*	0.49^a^	< 0.001*	0.21^b^	0.25	0.03^b^	0.85	-0.05^a^	0.75
**Adults (** ***n*** ** = 41)**										
**TUG (s)**	-0.38^a^	0.01*	-0.35^a^	0.02*	0,05^b^	0.98	0.16^b^	0.31	-0.24^b^	0.11
**5R-STS (s)**	-0.44^b^	< 0.001*	-0.48^b^	< 0.001*	0.09^b^	0.55	0.32^b^	0.03*	-0.13^b^	0.38
**Agility 4 × 10 m (s)**	-0.51^b^	< 0.001*	-0.47^b^	< 0.001*	-0.23^b^	0.14	0.04^b^	0.77	-0.12^b^	0.45
**CMJ (cm)**	0.70^a^	< 0.001*	0.71^a^	< 0.001*	0.37^b^	0.01*	-0.03^b^	0.82	-0.20^b^	0.19

HGS-A: Absolute handgrip strength; HGS-R: Relative handgrip strength; CC: Calf circumference; TUG: Timed Up and Go; 5R-STS: Five Repetition Sit-to-Stand Test; CMJ: Countermovement Jump; a: “r” value from Pearson’s Correlation Test; b: “r” value from Spearman’s Correlation Test; *:Significance level *p* < 0.05

Additionally, a univariate linear regression model was used to analyze the relationship between HGS-A, HGS-R, HGS-A asymmetries, and CC with various functional tests (Table 4). Significant associations (*p* < 0.05) were observed between HGS-A and HGS-R with functional tests across all age groups. The HGS-A was associated with the 4 × 10 m agility test in adolescents (β = -0.89; 95% CI: -1.76, -0.04) and the TUG test in adults (β = 2.12; 95% CI: 0.24, 3.99). In contrast, the percentage asymmetry of HGS-A showed associations in the adult group with the TUG test (β = 0.11; 95% CI: 0.06, 0.16), the 5R-STS test (β = 0.29; 95% CI: 0.15, 0.43), and the 4 × 10 m agility test (β = 0.29; 95% CI: 0.15, 0.43). An increase of 1 kg in HGS-A corresponded to a decrease of -0.09 s in the TUG test, -0.17 s in the 5R-STS test, -0.30 s in the 4 × 10 m agility test, and an increase of 0.36 cm in the CMJ in the total sample of participants. No statistical association (*p* > 0.05) was found between CC and any of the functional tests.

**Table 4:** Univariate linear regression model between muscle strength indicators and functional capacity in schoolchildren with intellectual disabilities, differentiated by age group

Functional Capacity	HGS-A (Kg)			HGS-*R*			Asymmetry HGS-A (Kg)			Asymmetry HGS-A (%)			CC (cm)			
	β (IC 95%)	*R* ^2^	*p*-value	β (IC 95%)	*R* ^2^	*p*-value	β (IC 95%)	*R* ^2^	*p*-value	β (IC 95%)	*R* ^2^	*p*-value	β (IC 95%)	*R* ^2^	*p*-value	
**Total (** ***n*** ** = 102)**																
**TUG (s)**	-0.09 (-0.14; -0.06)	0.21	< 0.001*	-6.47 (-8.97; -3.97)	0.20	< 0.001*	-0.13 (-0.44; 0.17)	0.00	0.39	0.03 (-0.00; 0.06)	0.02	0.08	-0.12 (-0.21; -0.02)	0.05	0.02*	
**5R-STS (s)**	-0.17 (-0.23; -0.09)	0.19	< 0.001*	-12.41 (-16.77; -8.05)	0.24	< 0.001*	-0.19 (-0.75; 0.36)	0.00	0.48	0.05 (-0.01; 0.11)	0.03	0.07	-0.12 (-0.29; 0.06)	0.01	0.19	
**Agility 4 × 10 m (s)**	-0.30 (-0.39; -0.22)	0.32	< 0.001*	-19.59 (-25.48; -13.7)	0.30	< 0.001*	-0.45 (-1.22; 0.33)	0.01	0.25	0.12 (0.04; 0.19)	0.08	< 0.001*	-0.20 (-0.45; 0.05)	0.02	0.11	
**CMJ (cm)**	0.36 (0.28; 0.45)	0.41	< 0.001*	24.65 (19.07; 30.23)	0.43	< 0.001*	0.51 (-0.30; 1.33)	0.01	0.21	-0.06 (-0.15; 0.03)	0.01	0.17	-0.11 (-0.38; 0.15)	0.00	0.39	
**Children (** ***n*** ** = 31)**																
**TUG (s)**	-0.34 (-0.49; -0.19)	0.66	< 0.001*	-11.60 (-18.64; -4.55)	0.53	< 0.001*	-0.08 (-0.63; 0.47)	0.05	0.76	0.00 (-0.06; 0.06)	0.03	0.96	-0.15 (-0.38; 0.06)	0.26	0.15	
**5R-STS (s)**	-0.47 (-0.83; -0.11)	0.44	0.01*	-20.15 (-35.34; -4.97)	0.45	0.01*	-0.17 (-1.29; 0.95)	0.05	0.75	-0.02 (-0.15; 0.10)	0.06	0.72	-0.10 (-0.58; 0.36)	0.00	0.64	
**Agility 4 × 10 m (s)**	-0.69 (-1.03; -0.36)	0.62	< 0.001*	-30.89 (-44.38; -17.41)	0.65	< 0.001*	0.83 (-1.32; 1.04)	0.04	0.81	0.09 (-0.03; 0.23)	0.26	0.15	0.15 (-0.34; 0.64)	0.11	0.52	
**CMJ (cm)**	0.61 (0.29; 0.93)	0.58	< 0.001*	27.56 (14.62; 40.50)	0.62	< 0.001*	-0.09 (-1.19; 1.00)	0.03	0.85	-0.02 (-0.15; 0.10)	0.07	0.68	-0.25 (-0.70; 0.19)	0.21	0.25	
**Adolescents (** ***n*** ** = 30)**																
**TUG (s)**	-0.09 (-0.15; -0.04)	0.56	< 0.001*	-4.76 (-8.21; -1.32)	0.47	< 0.001*	-0.33 (-0.74; 0.07)	0.30	0.10	-0.02 (-0.07; 0.02)	0.18	0.31	-0.06 (-0.21; 0.08)	0.16	0.37	
**5R-STS (s)**	-0.12 (-0.18; -0.06)	0.59	< 0.001*	-7.04 (-10.63; -3.45)	0.60	< 0.001*	-0.39 (-0.86; 0.07)	0.31	0.09	-0.02 (-0.07; 0.03)	0.17	0.37	-0.02 (-0.19; 0.14)	0.00	0.74	
**Agility 4 × 10 m (s)**	-0.26 (-0.37; -0.14)	0.66	< 0.001*	-9.69 (-17.41; -1.98)	0.43	0.01*	-0.89 (-1.76; -0.04)	0.37	0.04*	-0.05 (-0.16; 0.04)	0.21	0.25	-0.29 (-0.59; 0.01)	0.34	0.05	
**CMJ (cm)**	0.39 (0.19; 0.59)	0.60	< 0.001*	18.27 (5.83; 30.71)	0.49	< 0.001*	-0.09 (-1.64; 1.47)	0.02	0.91	-0.09 (-0.27; 0.07)	0.21	0.24	-0.28 (-0.62; 0.46)	0.05	0.75	
**Adults (** ***n*** ** = 41)**																
**TUG (s)**	-0.09 (-0.16; -0.03)	0.40	< 0.001*	-5.58 (-10.41; -0.75)	0.35	0.02*	0.62 (-0.19; 1.43)	0.23	0.13	0.11 (0.06; 0.16)	0.60	< 0.001*	-0.14 (-0.32; 0.03)	0.24	0.11	
**5R-STS (s)**	-0.17 (-0.28; -0.06)	0.43	< 0.001*	-12.70 (-20.21; -5.19)	0.48	< 0.001*	1.13 (-0.21; 2.47)	0.26	0.09	0.21 (0.14; 0.28)	0.66	< 0.001*	-0.13 (-0.44; 0.17)	0.01	0.38	
**Agility 4 × 10 m (s)**	-0.31 (-0.50; -0.12)	0.47	< 0.001*	-21.56 (-34.45; -8.67)	0.47	< 0.001*	-0.14 (-1.53; 3.19)	0.11	0.48	0.29 (0.15; 0.43)	0.55	< 0.001*	-0.19 (-0.72; 0.33)	0.12	0.45	
**CMJ (cm)**	0.39 (0.27; 0.53)	0.71	< 0.001*	27.30 (18.70; 35.89)	0.71	< 0.001*	2.12 (0.24; 3.99)	0.34	0.02*	-0.04 (-0.19; 0.09)	0.10	0.51	-0.28 (-0.72; 0.14)	0.20	0.19	

HGS-A: Absolute handgrip strength; HGS-R: Relative handgrip strength; CC: Calf circumference; TUG: Timed Up and Go; 5R-STS: Five Repetition Sit-to-Stand Test; CMJ: Countermovement Jump. β: Beta value; IC95%: Confidence interval; R^2^: Coefficient of determination: *: Significance level *p* < 0.05

## Discussion

The objective of this study was to determine the association between absolute and relative HGS, upper-limb strength asymmetries, and CC with the functional capacity of individuals with ID across different age groups. The findings revealed a significant association between HGS and asymmetries with functional capacity, but not with CC, in individuals with ID.

HGS-A and HGS-R are well-established as biomarkers of muscular strength, overall health, and functional capacity across the lifespan [9]. A comparative analysis with normative data from Chilean children without ID reveals a significant deficit of 31.31% and 27.77% in HGS-A and HGS-R, among children with ID [34] Similarly, adults with ID demonstrated a 46.90% reduction in HGS-A compared to their non-ID counterparts, according to the results of the present study. The diminished muscle strength observed in individuals with ID may be attributed to multifactorial influences, including non-adherence to recommended physical activity guidelines, a prevalence of sedentary behaviors, comorbid ID-related pathologies [35], pharmacological interventions [36], and reduced capacity for voluntary strength generation [37].

The study’s results affirm that enhanced HGS-A and HGS-R are associated with superior functional capacity across all examined cohorts. Previous research [10] corroborates these findings, indicating that elevated levels of maximal isometric strength, as measured by dynamometric assessments of HGS and trunk extensor strength, correlate with improved functional performance in field tests among adolescents with moderate ID. Consequently, handgrip dynamometry emerges as a valuable tool for assessing functional capacity in this demographic. The findings align with the inverse correlation between HGS-A and the time required to complete the TUG, 5R-STS, and 4 × 10 m agility tests, alongside a direct relationship with CMJ height. Diminished HGS has been linked to limitations in activities of daily living [11], reduced gait speed [38, 39] and an elevated risk of falls, as evidenced by prolonged durations in the 5R-STS test [40].

The study also reveals a significant association between greater HGS-A asymmetries and longer execution times in most functional tests across age groups. Asymmetries exceeding 10% are known to correlate with functional decline [13], and the average asymmetry values observed in children (16.7%), adolescents (12.9%), and adults (11.8%) surpass this threshold, potentially heightening the risk of functional deterioration. These asymmetries may result in decreased gait velocity [41], an increased risk of sarcopenia [42], frailty [43], and a higher prevalence of comorbidities in individuals with ID [44].

Therefore, early intervention strategies are imperative to mitigate functional decline, as asymmetry may represent an antecedent muscular function deficit that precedes reduced HGS. Among the main intervention methods that can help reduce asymmetries, strength training is one of the most recommended [45]. Typical children and adolescents respond acutely to strength training in several areas. In addition to increasing muscle mass [46]. the neuromuscular response provides adaptations in economy, balance, and higher-quality muscle recruitment [47].

In another way, CC is traditionally regarded as a nutritional indicator associated with sarcopenia, dynapenia, functional decline, mortality, frailty, and physical disability in older adults [38]. Although the study did not find significant associations between CC and functional performance, prior research in older populations suggests that larger CC correlates with enhanced functionality [39]. The lack of significant findings in this study may be attributed to the greater sensitivity of muscle strength as an indicator of physical condition fluctuations compared to muscle mass [48]. Thus, prioritizing muscle function preservation over morphological attributes such as muscle size is recommended as a preventive measure against dynapenia in individuals with ID [49].

Individuals with ID often exhibit neuromuscular imbalances that may contribute to greater HGS asymmetry. These imbalances can stem from atypical motor development, reduced muscle tone, and compromised coordination. While direct studies on HGS asymmetry prevalence in the ID population are limited, research in older adults has demonstrated that HGS asymmetry is associated with functional limitations. For instance, McGrath et al. [50] found that HGS asymmetry and weakness were linked to lower cognitive function in older Americans. Although this study does not focus on individuals with ID, it underscores the potential impact of HGS asymmetry on functional outcomes. HGS asymmetry may adversely affect mobility and coordination, particularly in individuals with ID. In the general population, HGS asymmetry has been associated with slower gait speed and poorer standing balance. Research indicates that older adults with significant HGS asymmetry had greater odds of slow gait speed and impaired balance. Given that individuals with ID often experience baseline motor coordination challenges, the presence of HGS asymmetry could further exacerbate difficulties in mobility and coordination, potentially increasing the risk of falls and reducing independence in daily activities [51].

Finally, this study’s limitations include its cross-sectional design and the use of convenience sampling, which may affect the generalizability of the results. Therefore, future research should include cohort studies and randomized controlled trials to analyze response patterns, sensitivity over time, and responsiveness to change.

Despite these limitations, the study’s strengths lie in its heterogeneous sample, which includes children, adolescents, and adults, thus providing a comprehensive overview of the school cycle of Chilean individuals with ID. This research contributes valuable data on HGS and functionality in schoolchildren with ID, offering easily measurable indicators related to physical capacity components that are often compromised in this population. Notably, this study is among the first to investigate CC as a potential predictor of functional capacity in individuals with ID, an area that has been underexplored in existing literature. While the current study did not find significant results regarding CC, it establishes a foundation for future investigations to further explore this parameter’s role in functional capacity among individuals with ID.

Future research proposals should consider the investigation of absolute and relative HGS, as well as absolute asymmetries and functional capacity, taking into account sex differences, nutritional status, IQ level, specific syndromes associated with ID, and the influence of cardiorespiratory capacity on functional performance.

The results have practical implications, particularly in integrating strength training into the routine physical activity of children, adolescents and adults with ID. This approach can help address the observed disparities in muscle strength and functional performance. Regular engagement in strength training exercises can enhance neuromuscular function, improve balance and coordination, and promote greater independence in daily activities. Furthermore, strength training can play a critical role in reducing the prevalence of sedentary behaviors and encouraging an active lifestyle, which is vital for long-term health and well-being.

## Conclusion

Our findings demonstrate that both HGS-A and HGS-R, are key predictors of functional capacity in individuals with ID. These results underscore the critical need for early implementation of muscle strength training programs, beginning in the school years, to preserve and enhance functional capacity in this population. Such programs should incorporate diverse, evidence-based training methodologies tailored to the specific needs of individuals with ID, serving both preventive and rehabilitative purposes. Beyond physical benefits, strength training interventions can foster greater autonomy, promote overall well-being, and improve quality of life, reinforcing their importance as a fundamental component of health promotion strategies for this population.

## Data Availability

The datasets generated and/or analyzed during the current study are not publicly available due the terms of consent/assent to which the participants agreed but are available from the corresponding author on reasonable request. Please contact the corresponding author to discuss availability of data and materials.
